# The complete mitochondrial genome sequence and gene organization of *Ambassis gymnocephalus* (Perciformes, Ambassidae)

**DOI:** 10.1080/23802359.2017.1365636

**Published:** 2017-08-28

**Authors:** Yao Dong, Chunyan Ma, Hongyu Ma, Fengying Zhang, Liyan Qu, Wei Chen, Lingbo Ma

**Affiliations:** a Key Laboratory of East China Sea and Oceanic Fishery Resources Exploitation, Ministry of Agriculture, East China Sea Fisheries Research Institute, Chinese Academy of Fishery Sciences, Shanghai, China;; b College of Fisheries and Life Sciences, Shanghai Ocean University, Shanghai, China;; c Guangdong Provincial Key Laboratory of Marine Biology, Shantou University, Shantou, China

**Keywords:** Illumina sequencing, *Ambassis gymnocephalus*, mitogenome

## Abstract

In this study, we firstly obtained the complete mitochondrial genome DNA sequence of the *A*mbassis *gymnocephalus*. Its length is 17,388 bp, containing 13 protein-coding genes, 2 rRNAs, 22 tRNAs and a large noncoding region. The overall length of protein coding genes is 11,448 bp, including A (26.75%), G (15.20%), T (26.44%) and C (31.61%). The A + T content of control region (1555 bp) is 60.45%. The protein-coding gene COI is started by GTG and ended by AGA. From the phylogenetic tree, we find *A. gymnocephalus* and *Oreochromis niloticus* has the closest relationship among 21 related species. This work will provide some valuable information for further studies on *A. gymnocephalus* and the family Ambassidae.

*Ambassis gymnocephalus*, a member of Perciformes, broadly distributes in India, Indonesia, Philippines, Vietnam and China. *A. gymnocephalus* plays an important role on the aquatic ecosystem and is regarded as an index for evaluating species diversity and environmental change (Martin 1989; Tse et al. 2008). Mitochondrial DNA plays an important role on the studies of population genetics, evolutionary and phylogenetics (Avise et al. 1984; Zhong et al. 2013; Xia et al. 2015). To date, there was no introduction about the complete mitochondrial genome of the family Ambassidae. In this study, we obtained the complete mitochondrial genome DNA sequence of the *A. gymnocephalus* by Illumina sequencing and traditional Sanger sequencing, determined its mitochondrial genome structure and described its genome organization, gene arrangement and codon usage (Accession number KX621134). *A. gymnocephalus* individuals were collected from Guangxi Province of China (47°50′1″N; 126°28′32″E). After morphological identification, the specimens were stored in 95% ethanol until used. Muscle tissues were sampled and genomic DNA was extracted using Animal Genomic DNA Extraction Kit (TIANGEN, Beijing, China) following the operation manual. The DNA quality was assessed by 1.2% agarose gel and DNA was stored at −20 °C. The length of the complete mitochondrial genome DNA sequence of the *A. gymnocephalus* is 17,388 bp including 13 protein-coding genes, 2 rRNAs, 22 tRNAs and a large noncoding region between genes tRNA^Pro^ and tRNA^Phe^ with a high A + T content as a putative control region.

The overall base composition of the genome was 28.34% for A, 16.45% for G, 25.88% for T and 29.32% for C. The overall length of protein coding genes is 11,448 bp. Two types of start codon (GTG and ATG) were employed. Four types of stop codon were employed (TAA, TAG, AGA and (T−)). The control region (1555 bp) had the highest A + T content (60.45%) and the protein coding genes had the lowest A + T content (53.19%).

The phylogenetic tree of *A. gymnocephalus* among closely related 21 fishes was reconstructed based on the maximum likelihood (ML) method. It (Figure 1) suggests that *A. gymnocephalus* has the closest relationship with *Oreochromis niloticus*. This study will provide some useful information for studies on population genetic structure, evolution and conservation genetics of *A. gymnocephalus* and other related species.

**Figure 1.**
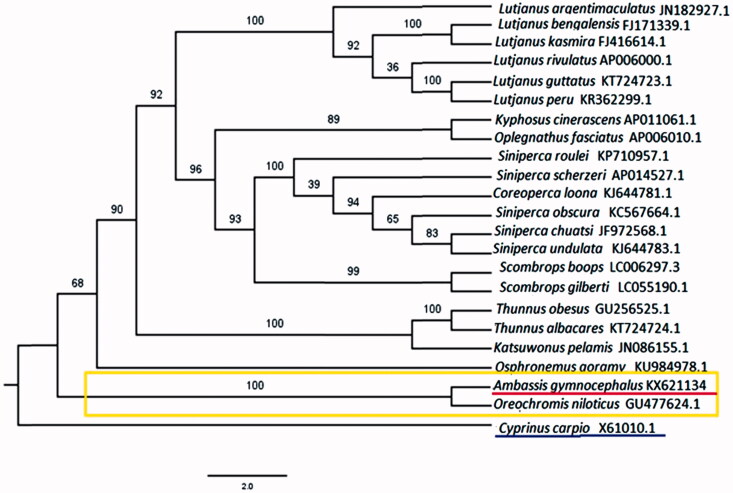
Maximum-likelihood tree of *Ambassis gymnocephalus* and 21 other species with NCBI accession number.
